# Suicidal Autointegration of *Sleeping Beauty* and *piggyBac* Transposons in Eukaryotic Cells

**DOI:** 10.1371/journal.pgen.1004103

**Published:** 2014-03-13

**Authors:** Yongming Wang, Jichang Wang, Anatharam Devaraj, Manvendra Singh, Ana Jimenez Orgaz, Jia-Xuan Chen, Matthias Selbach, Zoltán Ivics, Zsuzsanna Izsvák

**Affiliations:** 1 Max-Delbrück-Center for Molecular Medicine (MDC), Berlin, Germany; 2 State Key Laboratory of Genetic Engineering and Ministry of Education Key Laboratory of Contemporary Anthropology, School of Life Sciences, Fudan University, Shanghai, China; 3 Paul-Ehrlich-Institute, Division of Medical Biotechnology, Langen, Germany; University of Utah School of Medicine, United States of America

## Abstract

Transposons are discrete segments of DNA that have the distinctive ability to move and replicate within genomes across the tree of life. ‘Cut and paste’ DNA transposition involves excision from a donor locus and reintegration into a new locus in the genome. We studied molecular events following the excision steps of two eukaryotic DNA transposons, *Sleeping Beauty* (*SB*) and *piggyBac (PB)* that are widely used for genome manipulation in vertebrate species. *SB* originates from fish and *PB* from insects; thus, by introducing these transposons to human cells we aimed to monitor the process of establishing a transposon-host relationship in a naïve cellular environment. Similarly to retroviruses, neither *SB* nor *PB* is capable of self-avoidance because a significant portion of the excised transposons integrated back into its own genome in a suicidal process called autointegration. Barrier-to-autointegration factor (BANF1), a cellular co-factor of certain retroviruses, inhibited transposon autointegration, and was detected in higher-order protein complexes containing the *SB* transposase. Increasing size sensitized transposition for autointegration, consistent with elevated vulnerability of larger transposons. Both *SB* and *PB* were affected similarly by the size of the transposon in three different assays: excision, autointegration and productive transposition. Prior to reintegration, *SB* is completely separated from the donor molecule and followed an unbiased autointegration pattern, not associated with local hopping. Self-disruptive autointegration occurred at similar frequency for both transposons, while aberrant, pseudo-transposition events were more frequently observed for *PB*.

## Author Summary

Transposons (“jumping genes”) are ubiquitous, mobile genetic elements that make up significant fraction of genomes, and are best described as molecular parasites. During ‘cut and paste’ transposition, the excised transposon relocates from one genomic location to another. Here we focus on the molecular events following excision of two eukaryotic DNA transposons, *Sleeping Beauty* and *piggyBac*. Both transposons are primarily used in a cellular environment that is different from their original hosts, thereby offering a new model to study host-parasite interaction in higher organisms. In the last decade, they have been developed into a technology platform for vertebrate genetics, including gene discovery, transgenesis, gene therapy and stem cell manipulation. Despite the wide range of their application, relatively little is known about their molecular mechanism in vertebrates. We show that these elements are not capable of self-avoidance, as a significant portion of the excised transposons integrates into its own genome in a suicidal process. Despite mechanistic differences, both transposons are affected similarly, and larger transposons are particularly vulnerable. We propose that transposons might recruit phylogenetically conserved cellular factors in a new host that protects against self-disruption. Suboptimal conditions in a new environment could generate abnormal, genotoxic transposition reactions, and should be monitored.

## Introduction

Mobilization of transposable elements (TEs) is a DNA recombination reaction that can occur either via RNA (retroelement/retrovirus) or DNA intermediates (DNA transposon). In non-replicative, ‘cut and paste’ DNA transposition, the excised transposon relocates from one genomic location to another. In contrast, the ‘copy and paste’ mobilization of a retroelement/retrovirus does not include the excision step, but the downstream events of retroviral integration are highly similar to DNA transposition [1] Many DNA transposons are bracketed by terminal inverted repeats (IRs) that contain binding sites for the recombinase, the transposase. The transposition process is catalysed by the transposase, and can be divided into four steps: (i) the transposase recognizes and binds to the ends of the transposon; (ii) the transposase and two transposon ends form a complex called synaptic or paired end complex; (iii) the transposon is excised from the donor site; and (iv) the excised transposon is transferred to a new location by the transposase reviewed in [2].

TEs are ubiquitous components of both prokaryotic and eukaryotic genomes [3] Even though TEs are best viewed as molecular parasites that propagate themselves using resources of the host cells, their long-term coexistence with their host has provided ample examples of mutual adaptation. The mobility of TEs is regulated by diverse molecular mechanisms, and can be achieved by self-limiting regulatory features intrinsic to the TE itself [4] or mechanisms provided by the host cell. For example, the RNA interference (RNAi) machinery in eukaryotes is probably the best-known cellular mechanism that evolved to control transposition [5], [6]. Notably, generally little is known about the regulation of DNA transposons in eukaryotes. Indeed, our understanding of the mechanisms and the regulation of transposition in eukaryotes are mostly based on assuming analogies to bacterial transposons [2], [7], [8].

In the last decade, the DNA transposition of *Sleeping Beauty* (*SB*), a resurrected fish transposon [9] was intensively studied [10]–[13]. Using *SB* as a model to study host-transposon interaction in eukaryotic cells, a series of evolutionarily conserved (from fish to human) cellular determinants has been identified. HMGB1, a non-histone chromatin factor, is required for synaptic complex formation during *SB* transposition [11]. Factors of the non-homologous-end-joining (NHEJ) pathway of double strand DNA break (DSB) repair, including Ku70 and the DNA-dependent protein kinase (DNA-PKcs) are required for *SB* transposition by acting at repairing the transposon excision sites [10]. Through its association with Myc-interacting zinc finger protein 1 (ZBTB17 or Miz1), the *SB* transposase down-regulates cyclin D1 expression in human cells, resulting in a cell cycle slowdown [12]. A temporary G1 arrest enhances transposition, suggesting that *SB* transposition is favoured in the G1 phase of the cell cycle, where NHEJ is preferentially active [10]. The HMG-box transcription factor HMGXB4 (HMG2L1), a component of the Wnt-signaling pathway is involved in a feedback regulation of *SB* transposase expression [13]. These studies indicate that eukaryotic transposons can participate in a complex interactive regulatory platform involving evolutionary conserved cellular mechanisms.

Although, *SB* is a relatively well-characterised eukaryotic transposon, one part of the transposition reaction, the step following excision but prior reintegration, is yet unexplored. In the process of productive transposition, the excised molecule integrates into a new genomic location. However, in principle, the excised transposon molecule could reinsert, in a self-disruptive process, into its own genome. This suicidal transposition event is called autointegration, self-integration or intramolecular transposition, and is well characterized in prokaryotes [14]–[16]. The best-understood example in bacteria is *Tn*10 transposition, in which regulation of transposition is a delicate interplay between the transposon and host-encoded factors [17]–[19]. These host factors, namely IHF (integration host factor), HU (heat unstable nucleoid protein) and H-NS (nucleoid structuring protein) are among the most important regulatory factors in *E. coli*. IHF and HU stimulate the early steps of transposition prior to excision of *Tn*10 [19]. However, if they remain associated with the transpososome (a DNA-protein complex minimally containing the excised transposon and the transposase), they promote autointegration [7]. By opposing the effects of IHF [20] and HU, H-NS inhibits autointegration and promotes productive transposition [18], [19]. In eukaryotes, autointegration was reported in *mariner* transposition [21], [22] Curiously, one third of the autointegration events mediated by *Mos1* (*mariner*) were recovered from non-canonical target sites [22]. Self-disruptive autointegration has also been observed during retroviral integration [23]–[25]. A host-encoded protein, barrier-to-autointegration factor (BANF1 or BAF) has been identified by its ability to protect retroviruses from autointegration [23].

Two observations suggest that, similarly to bacterial transposons and retroviruses, autointegration could be a significant factor affecting productive DNA transposition in eukaryotes as well. First, similarly to certain bacterial DNA transposons [26], [27], transposition of *SB* from a genomic locus frequently occurs into sites that are close to the donor locus [28]; this phenomenon is termed “local hopping”. Obviously, the transposon itself is the closest target to integrate. In *Tn*10 transposition, the host factor IHF promotes ‘target site channelling’ close to the IR of the transposon [19]. Second, larger transposons are expected to be particularly attractive targets for autointegration. Indeed, it has been observed that, similarly to certain bacterial TEs, longer elements of *SB* tend to transpose less efficiently [29], [30]. Thus, both ‘local hoping’ and size-sensitivity might be associated with vulnerability of *SB* transposition to self-integration.

In the present study, we investigated the post-excision fate of two DNA transposons, *SB* [9] and *piggyBac* (*PB*) [31] in vertebrate cells. Although, both *SB* and *PB* belong to the superfamily of DDE/D transposases, characterized by a highly conserved catalytic domain [1], they exhibit significant differences in their mechanisms of transposition [32], [33]. For example, the activity of *SB* is essentially restricted to vertebrates [29], [34], with the exception of a chordate, *Ciona intestinalis* [35]. By contrast, *PB* seems to have an extremely wide host range as it can transpose in insects as well as in human cells [36]–[38]. In comparison to *PB*, *SB* was reported to exhibit a much stronger ‘local hopping’ phenotype [39], [40]. Furthermore, *SB*, but not *PB* was reported to be sensitive to the size of the mobilized element. Specifically, the transposition of *PB* was reported to be independent on the size of the element below 14 kb [41].

Importantly, both *SB* and *PB* are valuable genomic tools for genome manipulation [42], and mostly used in heterologous cellular environments, thereby offering a unique opportunity to investigate various survival strategies of DNA elements in eukaryotes. Indeed, we can model how these elements behave in naïve genomes, and adapt to their new environment. We have used a simple experimental setup, i. e., transfection into cultured cells to monitor the process of establishing a host-parasite relationship in a heterologous environment. This strategy identified BANF1 as a host-encoded factor influencing this process. We propose that deciphering the mechanism and regulation of transposon reactions and translating this knowledge can be effectively used to derive transposon-based genetic tools for genome manipulation or for gene therapy.

## Results

### Self-destructive autointegration events mediated by the *Sleeping Beauty* transposase

**Figure 1:**
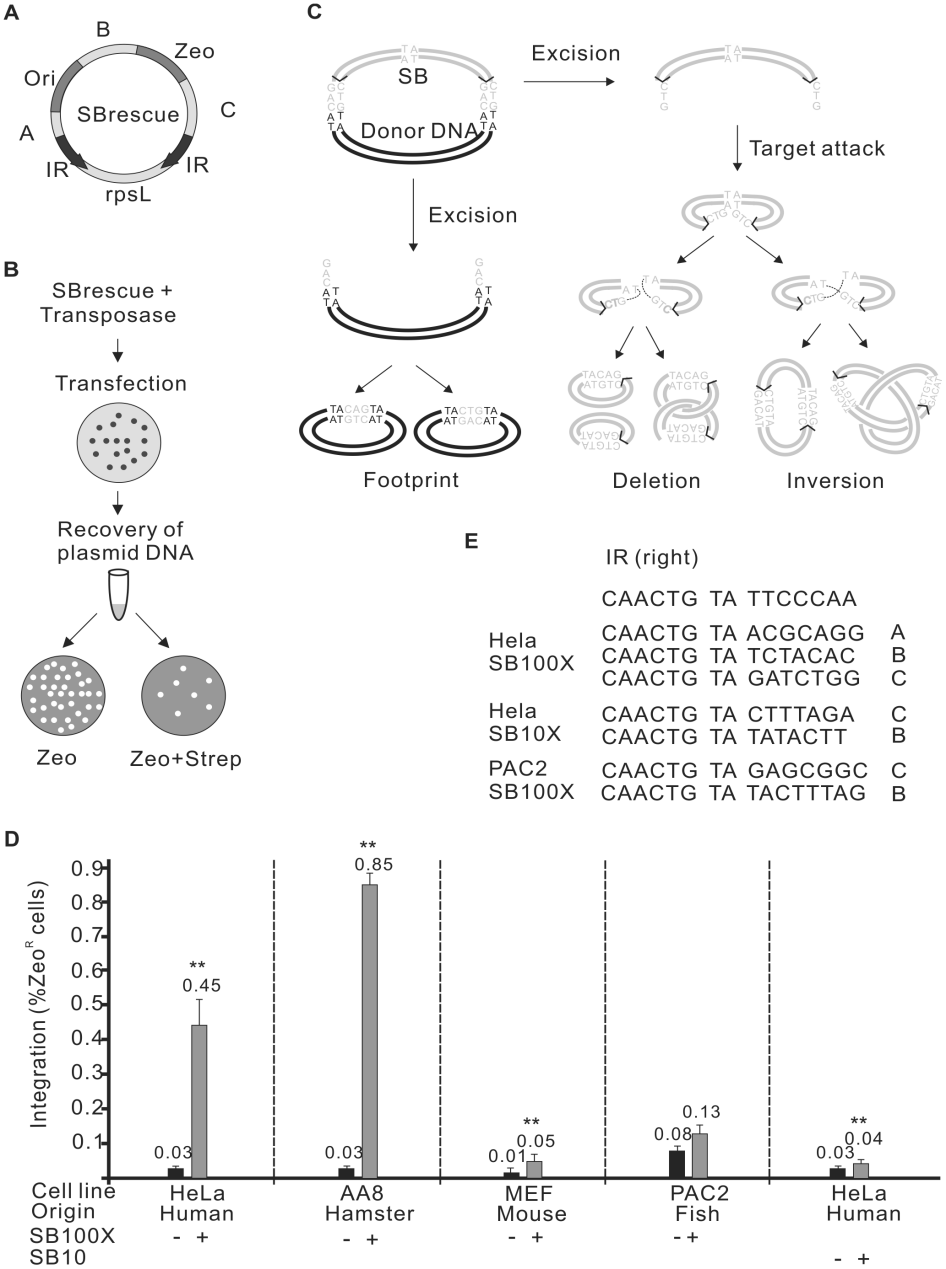
Autointegration of *SB* transposon. A. The structure of the *SBrescue* construct. The *SBrescue* contains an *SB* transposon carrying an origin of replication (Ori) and a zeocin gene (*Zeo*). The backbone DNA encodes a streptomycin sensitive gene rpsL (gray box). Bacteria carrying autointegration products with intact Ori and *Zeo* and integrations disrupting rpsL function (regions of A, B, C, IR and rpsL) could be rescued following double antibiotic selection of zeocin and streptomycin. Black arrow: inverted repeat (IR). B. Flowchart of the autointegration assay. *SBrescue* and helper plasmids encoding for the transposase are cotransfected into cells. Two days postransfection, low-molecular weight (plasmid) DNA is recovered from cells and transformed into bacteria. Bacteria were subjected to a selection of either zeocin or a double selection of zeocin/streptomycin. Frequency is calculated as zeo^R^/Strep^R^ normalized by zeo^R^. The assay would capture circularized molecules generated in eukaryotic cells, while linear DNA degrades in bacteria. C. A model of *SB* autointegration. The excision and reintegration steps of autointegration are similar to canonical transposition. Excision; The transposition initiates with a staggered cut. The *SB* transposon (gray lines) is separated from the donor DNA (black lines) by the transposase. Autointegration; the transposon attacks a target site (TA) within the transposon. The autointegration products can be either two deletion circles (left) or inversion products, knotted or unknotted (right). For details see [26]. In the inversion products the orientation of the IRs would be different from the donor substrate and would contain two ends of the transposon and target site duplications. The host DNA repair machinery would repair the single stranded gaps at the integration site and the double-strand breaks at the excision site [10]. The excision site repair products (also called “footprints”) can be either CAG or CTG (gray). The backbone DNA does not have Ori and would exist only transiently in bacteria. D. Frequency of *SB* autointegration events in various vertebrate cells HeLa (human), AA8 (Chinese hamster), MEF (mouse embryonic fibroblast) and PAC2 (zebrafish) cells using either *SB100X* [44] or *SB10* [9] transposases. The statistical significance of differences is shown by asterisk above the bars, **P<0.01. E. Confirmation of autointegration events by sequencing the *de novo* target sites. Sequences from the donor construct (bold) right-IR (6 bp), TA target sites (bold, italic) and 3′ flanking DNA (7 bp) are shown. The location of the targeted region is shown in the right side.

To detect and characterise potential autointegration products, the following assay system was established. The test construct, *SBrescue*, is a plasmid comprising a replication origin (Ori) and an antibiotic resistance cassette for zeocin (*Zeo*) located between the IRs of the transposon (Figure 1A). Outside of the transposon *SBrescue* contains the rpsL gene rendering bacteria sensitive to streptomycin [43]. *SBrescue* and the helper plasmid encoding for the transposase are co-transfected into cells. Plasmid DNA is recovered from the cells two days post-transfection and transformed into *E. coli*. Bacteria are subjected to double antibiotic selection of zeocin and streptomycin (Figure 1B). Following transposon excision and circularization of the excised transposon, the rpsL is lost, thereby rendering bacteria Strep^R^ (Figure 1B). Autointegrative transposition events can be rescued in the form of either two deletion circles or a single inversion circle, depending on the topology of the strand attack (Figure 1C). The assay can detect autointegration events occurring into regions designated A, B, C and IR (Figure 1A). In addition, integration events into the rpsL gene would render bacteria resistant to streptomycin and recovered by the assay. In contrast, autointegration events into *Zeo* or Ori would not be detectable with the assay system, because these regions are required for plasmid propagation and maintenance.

To identify conditions affecting autointegration of *SB*, the following factors were considered: (a) cell type specificity; (b) transposase activity; (c) target site distribution; (d) the size of the transposon; (e) host-transposon interaction. First, *SBrescue* was introduced into human HeLa cells with or without a helper plasmid expressing the hyperactive *SB100X* transposase [44] (Figure 1B). Compared to the control (0.03%, 1.19×10^3^/3.88×10^6^), significantly elevated numbers (0.45%, 4×10^3^/9.09×10^5^) of Zeo^R^/Strep^R^ bacterial colonies were observed when *SB100X* transposase was present in the experiments (Figure 1D). To characterize potential autointegration events and map the transposon insertion sites, the recovered products were subjected to DNA sequencing. Sequencing data confirmed that similarly to productive transposition, the autointegration events of *SB* transposition were targeted into TA dinucleotides within the mappable A, B, C and IR regions of the transposon (Figure 1E). To investigate if cellular factors in various vertebrate species might differentially promote or protect against autointegration of *SB*, the assay was performed in cultured cells of different origin, including AA8 (Chinese hamster, ovarian; 0.85% vs 0.03%, 1.51×10^3^/1.77×10^5^ vs 476/5.52×10^6^), MEF (mouse, embryonic fibroblast; 0.05% vs 0.01%, 8.06×10^3^/1.71×10^7^ vs 332/9.54×10^5^) and PAC2 (zebrafish, fibroblast; 0.13% vs 0.08%, 1.03×10^3^/8.42×10^5^ vs 770/9.69×10^5^) cells (Figure 1D). Our results revealed that the *SB*-mediated autointegration events were detectable in all tested cell lines, including fish, the natural cellular environment of *SB* (Figure 1D). Similarly to productive transposition, the frequency of autointegration varied in the different cell types [29]. The highest frequencies of autointegration were detected in HeLa and AA8 cells that generally support efficient transposition [29], suggesting that the frequency of autointegration was primarily dependent on the activity of the transposase, rather than the cell type (Figure 1D). Indeed, compared to the original *SB10* transposase [9], autointegration by the hyperactive *SB100X* transposase [44] was higher by one order of magnitude in human HeLa cells.

**Figure 2:**
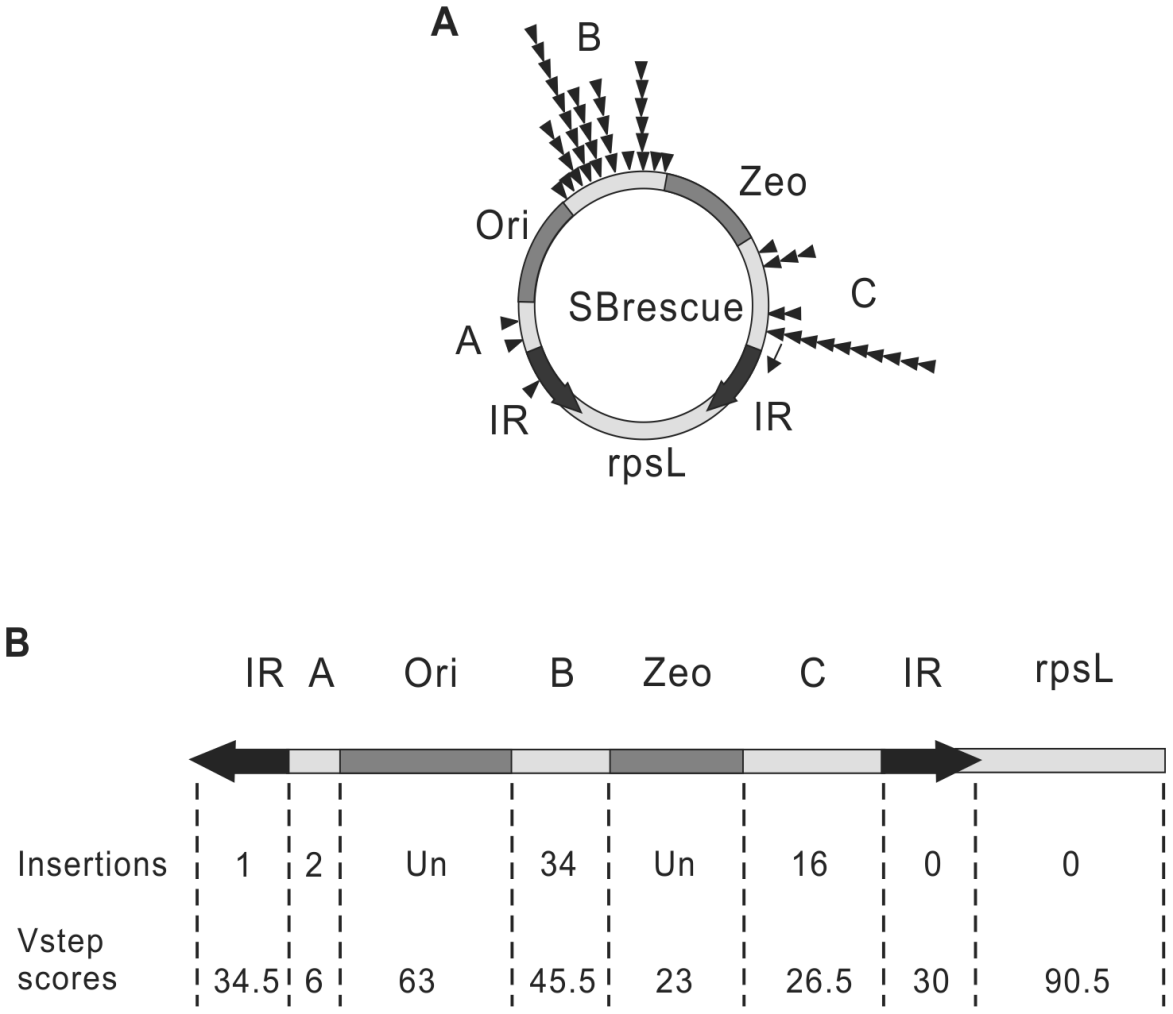
Comparing autointegration profile to the predicted, close-to-random target site distribution of *SB* transposition. A. Distribution of 53 *de novo* autointegration events (triangles) detected by the assay shown in (Figure 1B). Autointegration products were isolated from individual bacterial clones, sequenced and mapped to the *SBrescue* construct. The thin arrow indicates the location of the sequencing primer on the left IR. B. Comparison of the predicted and experimental insertion events. The *SBrescue* construct is shown in a linear mode. The *SB Vstep* scores and experimental insertion events were shown below. Un, undetectable.

Remobilization of the *SB* transposon from a genomic donor site exhibits a significant bias toward the donor locus (local hopping) [32]. Similarly, the reintegration of *Tn*10 transposons is not unbiased and targeted to the IRs of the transposon during autointegration, referred as ‘target site channelling’ [19]. In contrast, when launched from an extrachromosomal donor molecule, the genomic distribution of *SB* insertion sites is fairly random [45]–[47]. Target site selection during transposition of *SB* from an extrachromosomal plasmid is primarily determined on the level of DNA structure, as insertion sites tend to have a palindromic pattern and a bendable structure [45]. Accordingly, the insertion profile of the *SB* transposon can be modelled by determining the DNA-deformability scores, called *Vstep* for each potential TA target site, using the software *ProTIS* [48]. To determine the autointegration profile of *SB*, *Vstep* values were generated for the mappable regions of *SBrescue* and the observed insertion frequencies were compared to the calculated *Vstep* values (Figure 2B). Altogether, 53 autointegration products were identified and mapped to the regions of IR, A, B and C. Most of the autointegration events occurred into region B that is farther away from the IRs, and relatively few into regions A and C that are closer to the transposon ends (Figure 2). In regions B and C, there was a correlation between insertion frequencies and *Vstep* scores (Figure 2B). These results suggest that similarly to transposition from an extrachromosomal donor, insertion site selection during autointegration of *SB* is largely independent from the donor site and did not exhibit ‘target site channelling’ close to the IRs of the transposon. On the contrary, despite of the predicted high *Vstep* score, only a single insertion event was recovered from the IRs (Figure 2), suggesting that the transposon ends of *SB*, embedded in a paired end complex are limited in their abilities to target the IRs or sites close to the IRs during autointegration. Due to the linkage, the autointegration of *SB* was primarily intramolecular, and no insertions were detected from the rpsL region. Thus, the transposon was fully excised from the flanking donor DNA prior its integration into a new site.

### Autointegration properties of the *piggyBac* transposon and single-ended transposition

**Figure 3:**
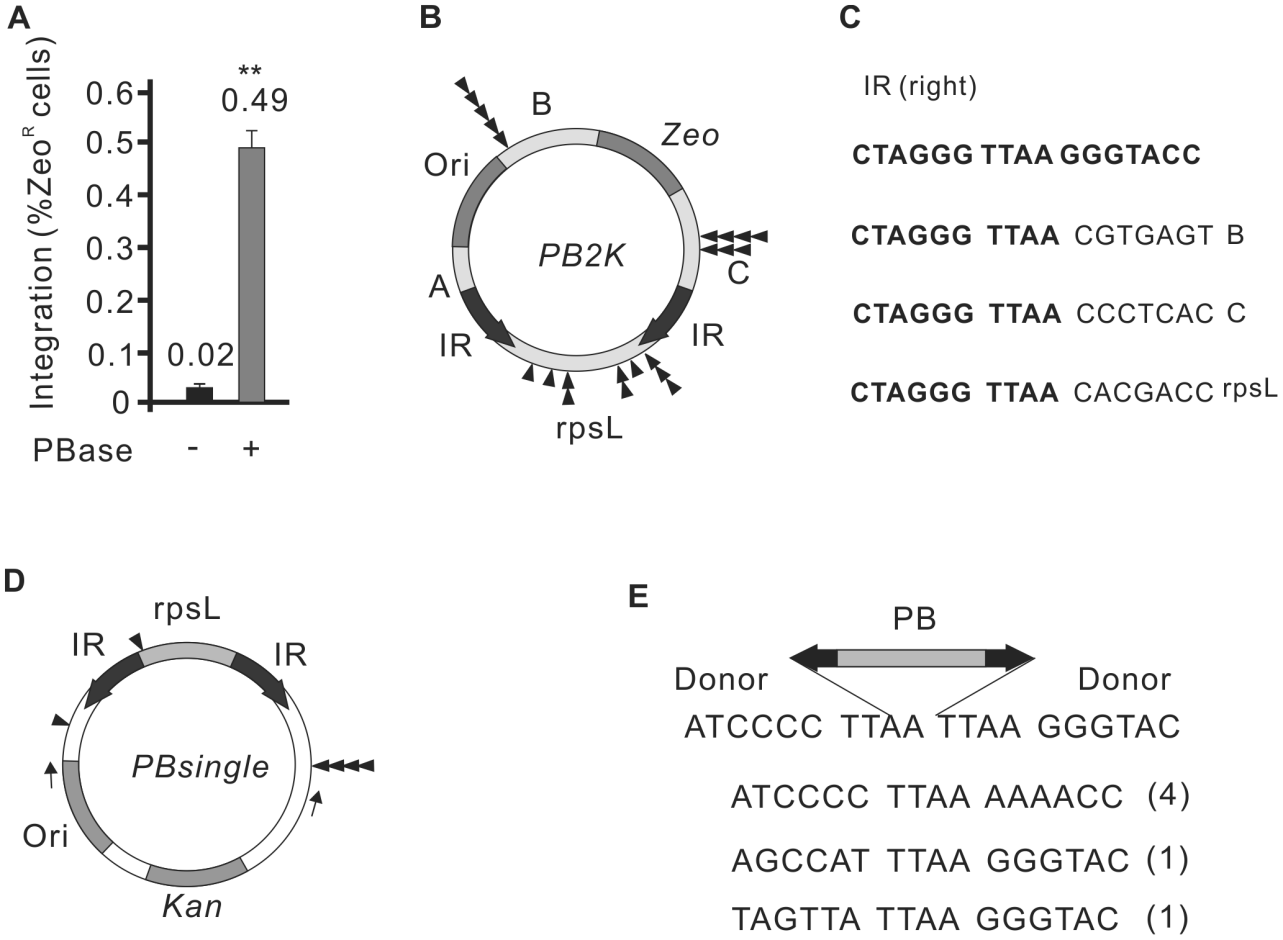
Autointegration properties of *PiggyBac* transposition. A. Frequency of *PB* autointegration events in HeLa cells using the PB2K construct. PBase, m*PB* transposase [50]. B. The structure of the *PB2K* construct. For explanation, see Figure 1A. Distribution of *de novo PB* insertions indicated by black triangles (n = 22) on the *PB2K* construct. C. Sequence of three (3/22) representative single-ended transposition events mapped to the B, C and rpsL regions of *PB2K*. Sequences flanking the right inverted repeat of the *PB* transposon in *PB2K*. Original sequences (bold); *de novo* integration events (normal); target site of PB transposition, TTAA (italic). D. Distribution of six single-ended transposition events on the *PBsingle* construct. *Kan*: kanamycin resistant gene (*Kan*). Dark bars indicate the control experiment with only transposon vector; light bars indicate the experiment with both transposon vector and transposase expressing vector. E. Sequence of the six individual single-ended transposition shown on Figure 3D. The *PB* transposon is shown as a two-headed arrow, representing the IRs (black). Frequencies are shown in parentheses.

Next, we tested whether self-destructive autointegration could also occur during *PB* transposition. We have used a transposon donor construct that is identical to *SBrescue*, except that the *SB* IRs were replaced by *PB* IRs [49] (*PB2K* in Fig. 3B), together with a mouse codon-optimized *PB* transposase (*mPB*) [50]. As shown in Figure 3A, autointegration of the *PB* transposon occurred at frequencies comparable to SB100X (0.49%, 3.2×10^4^/6.4×10^6^) in HeLa cells. As predicted and confirmed by DNA sequencing, autointegration of *PB* occurred into TTAA motifs, the canonical target site of *PB* [31] (Supporting Figure S1). Altogether, 23 integration sites were mapped and twelve were recovered from regions B and C (Figures 3B,C). However, unlike with *SB*, a significant number of integration events (48%, 11/23) mapped outside of the transposon, in the rpsL gene (Figures 3B,C). These non-canonical transposition events also targeted TTAA target sites, but involved only a single end of the transposon. The other IR was not separated from the donor molecule during the reaction. We refer to these non-canonical transposition events as single-ended transposition.

To investigate the phenomenon of single-ended transposition of *PB* further, a reciprocal construct, *PBsingle* was generated, where the *PB* transposon carried an rpsL gene (Figure 3D). In addition to single-ended transposition events detected by *PB2K*, the *PBsingle* assay system was suitable to capture various deletion products (Supporting Figure S2). Bacteria that gained Strep^R^ could report on (i) double-ended excision products, (ii) single-ended integration events into either rpsL or (iii) the vector sequence flanking the transposon. The autointegration assay was performed as shown in Figure 1B, except bacteria were exposed to double selection of kanamycin and streptomycin. To capture single-ended events, 336 transposition products were pre-filtered by colony PCR, using primers flanking the *PB* excision site. Canonical excision products would appear as uniformly sized PCR products, while size difference would report on either single-ended transposition or non-transposase-mediated small deletions/insertion events generated by DNA repair. 31/336 pre-filtered PCR products were analysed further by DNA sequencing, and six out of 31 (19%) products were clearly generated by *PB* transposase-mediated, single-ended transposition that occurred into TTAA either inside or outside of the transposon (Figures 3D and 3E).

### Bimolecular transposition

In the ‘single-ended’ transposition of *PB*, only one of the IRs was mobilized. Still, true single ended events, when the second IR is not involved in any of the steps of transposition, cannot be convincingly demonstrated. In fact, alternative mechanisms can generate similar, hard-to distinguish products. For example, the canonical transposition reaction might fail at the final step, and only one end of the transposon is transferred (lariat model), (Supporting Figure S2). Aberrant transposition might also occur by a mechanism that involves pseudo or cryptic sites mistakenly recognized as IRs. In addition, the ends of the transposon can also be derived from two separate molecules [51] (bimolecular transposition).

**Figure 4:**
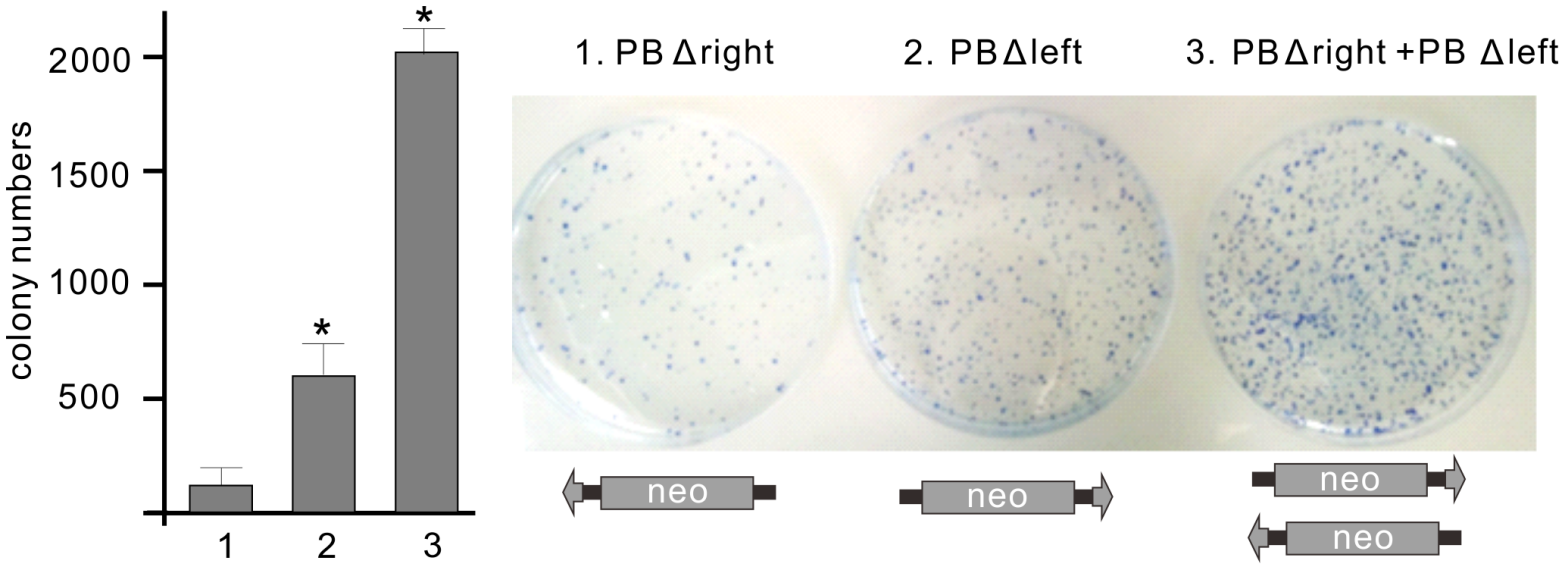
Bimolecular transposition events generated by *PB*. Transposition assay was performed by using ‘solo’ transposon substrates, either alone or mixed in equimolar ratios, in the present of the mPB transposase. The statistical significance of differences is shown by asterisk above the bars, *P<0.05. Molecular analysis identified no transposase-mediated integration events in the resistant colonies using *PBΔright* (background). See also Table 1.

To explore the scenario of bimolecular transposition, truncated ‘solo’ transposons were generated. ‘Solo’ substrates, lacking either the left (*PBΔleft; SBΔleft*) or the right IRs (*PBΔright; SBΔright*) were tested in a cell culture-based transposition assay [9]. Molecular analysis of the resistant colonies revealed that neither *PBΔright* nor *SBΔleft* supported transposition (Table 1). In contrast, the analysis confirmed transposase-mediated transposition of the ‘solo’ substrates, *PBΔleft* (4.6%) and *SBΔright* (0.56%) [52] (Table 1), indicating that both transposases are capable of utilizing ‘solo’ substrates. In either cases, the IRs of the ‘solo’ transposons were properly integrated into respective target sites (Supporting Test S1). Notably, in clone *PBΔleft#8*, we have identified a second right IR integrated into a same genomic locus, confirming that the transposase used the two IRs from separate molecules (Supporting Text SF1). As ‘solo’ transposition occurred ∼8-fold more frequently for *PB*, we monitored the *PB* system further in the ‘solo-mixing’ experiments. In this strategy, the *PBΔleft* and *PBΔright* constructs were transfected either alone or mixed in equimolar ratios, and tested in the colony forming, transposition assay. If transposition utilizes the IRs from separate molecules, one would expect elevated colony numbers when either *PBΔleft* or both ‘solo’ substrates are present in the assay, compared to *PBΔright* that does not support transposition alone (Table 1). The higher number of resistant colonies in the respective experiments indicated that the transposase was able to utilize the IRs from different copies of the transposon, supporting the bimolecular model (Figure 4).

**Table 1:** Transposase-mediated integration events of ‘solo’ substrates.

	*PBΔright*	*PBΔleft*	*SBΔleft*	*SBΔright*
Substrate integration frequency	2.6%	13.6%	3.4%	7.9%
Analysed number of resistant colonies	30	41	30	70
Transposase-mediated ‘solo’ integrations	0	14	0	5
Transposase-mediated ‘solo’ integration (%)	ND	4.6%	ND	0.56%

HeLa cells were co-transfected with the ‘solo’ transposon constructs in the present of either mPB or SB100X transposases, while a catalytically inactive *SB* transposase, D3 was used as a control. Frequency of substrate integration was calculated as a ratio of colony numbers in the presence vs absence of transposases. Colonies were picked and analysed for transposase-mediated integration events. Transposase-mediated integration is defined when the IR of the transposon is integrated into a respective target site in the genome (see also Supporting Text S1). ND: not detected.

### Both *SB* and *PB* transposons are sensitive to the size of the transposon

**Figure 5:**
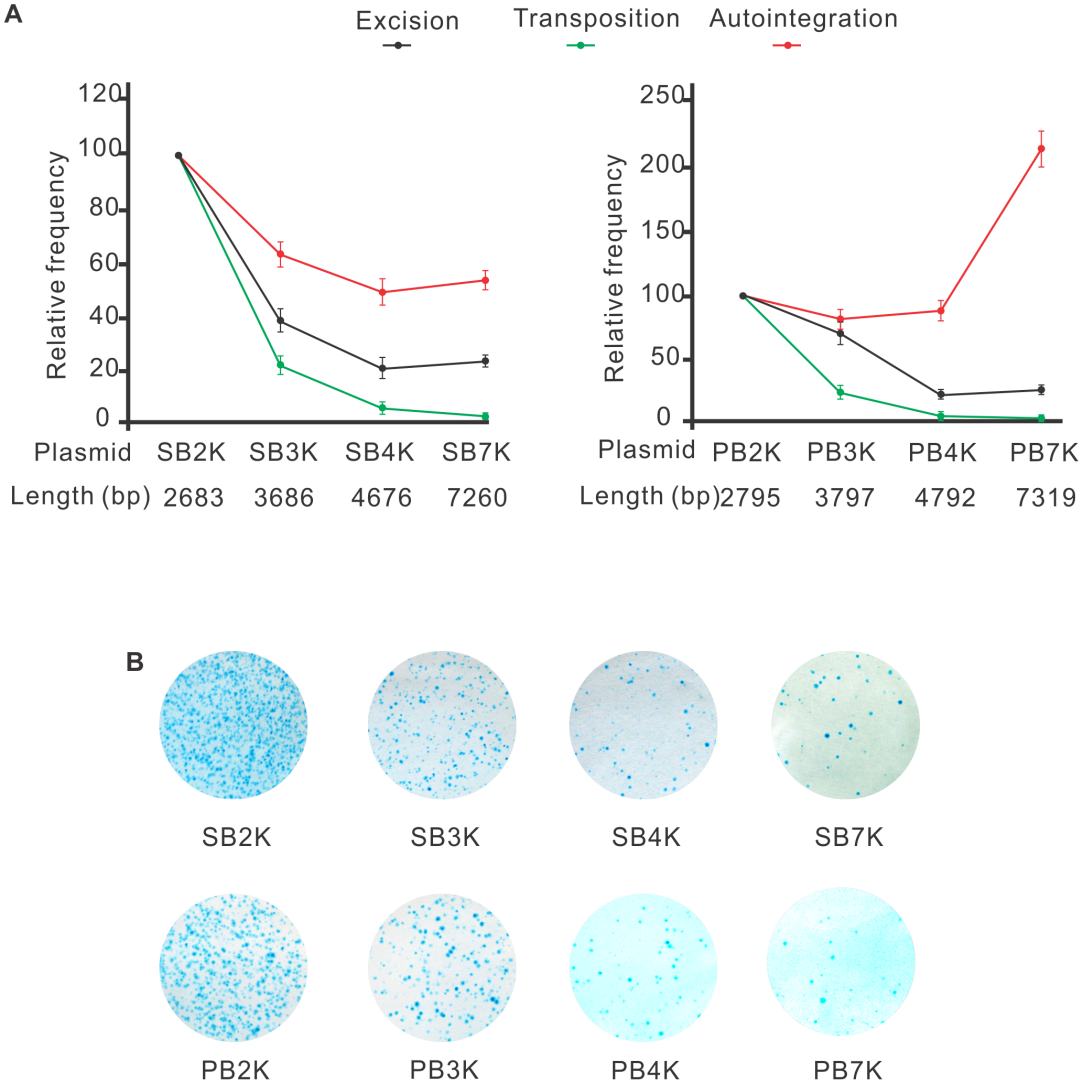
Both *SB* and *PB* transposons are sensitive to the size of the transposon. A. Excision, autointegration and transposition profiles of *SB* (left panel) and *PB* (right panel) transposons. The name and the size of the various constructs are shown below the plots. The values using the smallest constructs (SB2K or PB2K) were set to 100% (n = 3). B. Transposition assay performed by using *SB* (upper panel) and *PB* (lower panel) transposon constructs of various sizes.

The efficacy of transposition was reported to depend on the size of the transposon [29], [30], [53]–[55]. One potential mechanism responsible for such size-dependence is that following transposon excision, self-disruptive autointegration competes with productive transposition. Since larger transposons have more target sites, they could be particularly attractive targets for autointegration. This hypothesis predicts that the size of the transposon does not affect the frequency of excision, but it shifts the ratio between autointegration and productive transposition. To test this assumption, a series of transposons of different size, ranging from 2679 bp to 7256 bp (*SB2K, SB3K, SB4K, SB7K*) and 2795 bp to 7319 bp (*PB2K, PB3K, PB4K, PB7K*) were generated for *SB* and *PB*, respectively. Frequencies of transposon excision, autointegration and productive transposition events were determined for the various transposons. Excision frequencies were estimated by quantitative PCR, autointegration was monitored as above. Productive transposition was determined in a cell culture-based assay [9]. Figure 5A shows that excision frequencies declined with increasing size, while autointegration frequencies elevated over 4 kb either moderately or sharply for *SB* and *PB* transposons, respectively (Figure 5A). Accordingly, productive transposition frequencies dropped with increasing size of both *SB* and *PB* transposons. These results indicated that the size of the transposon affected transposition already at the excision step, thereby arguing against the hypothesis of autointegration being the sole factor that compromises productive transposition with increasing transposon size. Nevertheless, autointegration contributes as an additive element to the less efficient transposition of long transposons. Surprisingly, the two transposons behaved similarly in all three assays (Figures 5A an 5B). Thus, in contrast to general assumptions, and similarly to *SB*, size affects *PB* transposition as well.

### Inhibition of autointegration by a cellular, barrier-to-autointegration factor

**Figure 6:**
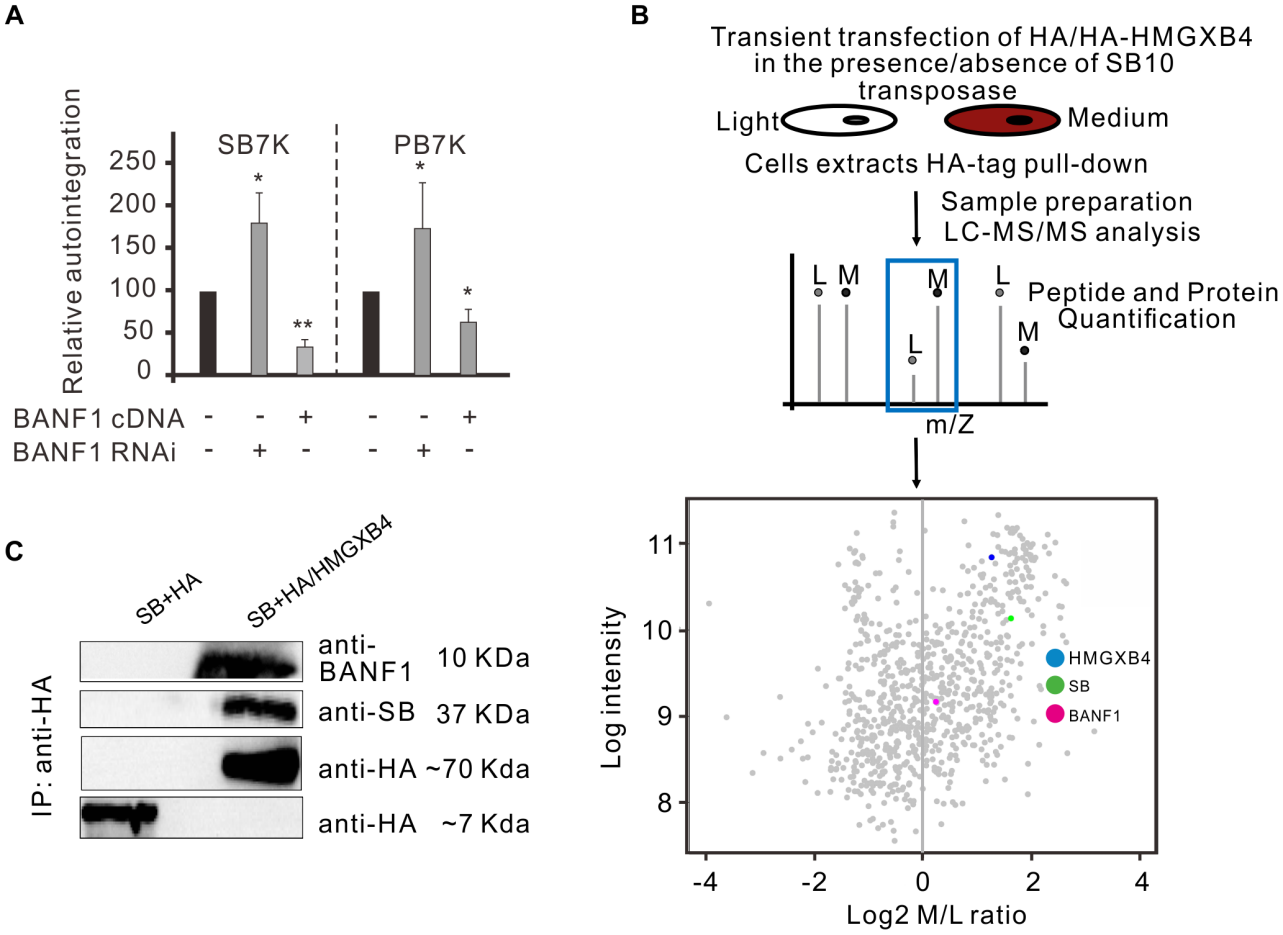
The cellular factor of BANF1 interferes with autointegration. A. Relative autointegration frequencies of *SB* (*SB7K*, left panel) and PB (*PB7K*, right panel) in HeLa cells, where BANF1 was either knocked-down or overexpressed. Knocking down of BANF1 stimulated, whereas overexpressing of BANF1 inhibited autointegration of both *SB* and *PB* transposons (n = 3). The statistical significance of differences is shown by asterisk above the bars *P<0.05. B. A SILAC pull-down experiment using anti-HA resin to investigate interaction partners of HMGXB4 in the presence/absence of *SB10* transposase in transiently transfected HEK293T cells. Schematic representation of the SILAC/pull-down experimental approach in which stable isotope labeled amino acids [Light (L) or Medium heavy (M)] are added in the form of medium supplement to culture HEK293T cells. Detection of interaction partners is performed by mass spectrometry. Scatter plot displays the normalized log2 SILAC ratio M/L values (X-axis) versus log2 intensity (Y-axis) of proteins detected in the interactome around HMGXB4^−^ in presence of the SB transposase. Each dot represents an individual protein, while their position indicates their abundance in the complex pulled down by the bait of HMGXB4. Proteins with a positive log2 SILAC M/L ratio, including BANF1 and *SB* are enriched in the protein complex around HMGXB4. C. Co-immunoprecipitation assay to investigate the interaction partners of HMGXB4, a physical interaction partner of the *Sleeping Beauty* transposase, SB10 [13]. *SB10* and HA-tagged HMGXB4 were transiently transfected into HEK293T cells (see Methods). In comparison to negative control, BANF1 and *SB* are enriched in the pull-down by HMGXB4-HA.

A cellular protein, BANF1 (BAF) barrier-to-autointegration factor was identified by its ability to protect retroviruses from autointegration [23]. BANF1 binds to double-stranded DNA, including freshly transfected, extrachromosomal plasmid DNA [56], in a non-specific manner [57], [58]. Thus, in principle, BANF1 could affect DNA transposition as well, between the molecular steps of excision and reintegration, when the transposon exists as an extrachromosomal molecule in the cell. To test this assumption, we asked if BANF1 could protect DNA transposons from autointegration. We addressed this question by monitoring autointegration events in HeLa cells, where BANF1 was either knocked-down or transiently overexpressed (Figure 6A). When BANF1 expression was knocked-down by RNA interference (Supporting Figure S3), the frequency of autointegration of *SB* was increased by two-fold compared to the control (Figure 6A, left panel). In contrast, BANF1 overexpression decreased the frequency of autointegration to one third (Figure 6A, left panel). Similar results were obtained by using the *PB* transposon (Figure 6A, right panel). No significant effect of BANF1 was observed at the excision step of *SB* transposition (not shown), suggesting the BANF1 acted specifically following excision.

In addition to BANF1, the effect of another host-encoded factor, the high-mobility group protein (HMGB1) was tested on autointegration. Similarly to BANF1, HMGB1 binds DNA in a non-specific manner [59]. In *SB* transposition, the transposase physically associates with HMGB1 and recruits it to the transposon DNA [11]. Autointegration was monitored in cells where HMGB1 was either transiently overexpressed or knocked-out [60]. Although, HMGB1 overexpression or deficiency was significantly affecting productive transposition [11], it had no detectable influence on autointegration (Supporting Figure S4). These results indicate that despite their similar non-specific DNA-binding activity, BANF1 and HMGB1 have a clearly distinct effect on DNA transposition.

Alternatively to a non-specific engagement, and similarly to retroviruses, BANF1 might be actively recruited to a preintegration complex of a transposon. In order to distinguish between these two scenarios, a high throughput immunoprecipitation experiment was designed to analyse a protein interactome forming around the *SB* transposase in mammalian cells. Affinity purification combined with mass spectrometry is a powerful strategy to detect protein-protein interactions among proteins in their native cellular environment [61]. This method is suitable to reveal the composition of entire protein complexes. If we use the analogy to retroviruses [62], one should keep in mind that even if BANF1 is recruited actively to the preintegration complex, it might not be recruited directly by the transposase. To distinguish true interaction partners from non-specific contaminants, we needed an easy-to-detect, confirmed interacting partner of the *SB* transposase as bait. We can readily monitor interactions of HMGXB4 (HMG2l1) with either the transposon or the transposase *in vivo* [13]. Thus, HMGXB4 was chosen as bait to analyse higher order complexes formed around *SB*. The experiments were run in parallel, in the presence and in the absence of the *SB* transposase. In the control experiment, it is not expected to detect interaction partners of the *SB* transposase. HEK293T cells were transiently transfected with HA-tagged HMGXB4 protein in the presence/absence of the *SB10* transposase [9]. A SILAC pull-down experiment was performed. This experimental strategy identified BANF1 as an interaction partner of HMGXB4^−^ in the presence, but not in the absence of the *SB* transposase (Figure 6B). The presence of BANF1 was also detectable when the bait, HMGXB4 was used in a co-immunoprecipitation assay (Figure 6C). This observation predicts that BANF1 can be actively recruited into a higher order protein complexes forming around the *SB* transposase in mammalian cells.

## Discussion

### Suicidal autointegration of *Sleeping Beauty* and *piggyBac* transposons

This study focuses on molecular events following the excision steps of two eukaryotic DNA transposons, *SB* and *PB*, derived from fish and insect genomes, respectively. The transposition reactions were performed in a heterologous host environment, phylogenetically distant from their natural hosts. The experimental setup mimics the scenario of introducing DNA transposons into a naïve eukaryotic host. We have shown that a significant portion of *SB* and *PB* transposon excision events is accompanied by suicidal integration into the transposon's own DNA. Although, different transposons may have different frequency of autointegration depending on the structure of the transpososome and the number of the integration target sites on the transposon, autointegration would influence the success of a transposon in a new environment. Neither *SB* nor *PB* was immune to the suicidal process of autointegration. Thus, in general, transposases/integrases in eukaryotes might not be able to distinguish between their own genome form foreign DNA. This would define autointegration as the lack of ability of self-avoidance upon integration. In contrast, certain prokaryotic transposons, including *Tn*7 and *Mu* exhibit ‘target immunity’ that prevents the transposon from transposing into its own genome [63], [64]. Both *Tn*7 and *Mu* avoid integration into DNA molecules that already have a copy of the transposon. As an alternative to self-encoded ‘target immunity’, some bacterial transposons and eukaryotic retroviruses recruit cellular host factors to protect against autointegration [19], [23]–[25]. In *Tn*10 transposition a host protein, histone-like nucleoid structuring (H-NS) plays a role in promoting intermolecular and supressing self-destructive intramolecular integration events [19]. Similarly, DNA transposons in eukaryotes might also capture cellular factors to protect their genome against autointegration. This strategy could defend the invading molecule and contribute establishing a stable host-transposon relationship.

### BANF1 interferes with self-destructive autointegration of *SB* and *PB* transposons in eukaryotes

BANF1 is involved in several critical processes, including host defence [65], [66]. The usual mode of BANF1 is repressive, due to its propensity to coat DNA. For example, BANF1 acts as a potent inhibitor of virus replication, defending against poxvirus invasion [67]. Intriguingly, and in contrast to its original function in host defence, BANF1 is piggybacked by various retroviruses to protect their viral genome against autointegration. BANF1 inhibits autointegration of the Moloney Murine Leukemia retrovirus, MoMLV [23], [68], [69] or HIV-1 [62]. By physically protecting the retrovirus, BANF1 promotes productive viral integration into the host genome [62]. In our experimental setup, BANF1 was influencing the fate of the excised molecules of two DNA transposons of different origin, *SB* and *PB*. Thus, in addition to its reported activity to bind freshly transfected DNA [56] or retroviral cDNA [23], BANF1 might influence the fate of DNA transposons as well. An important ramification of utilizing phylogenetically conserved cellular proteins by transposons might be the ability to survive and establish stable host-parasite relationship in a heterologous host environment. Accordingly, in addition to its role in *Tn*10 transposition, H-NS was reported to selectively bind the transpososomes of *Tn*5, and is likely to modulate many other transposition processes in Gram-negative bacteria [70].

*SB* and *PB* are members of the superfamily of DDE/D transposases and retroviral integrases, utilizing the same strategy for target joining. Still, how reasonable it is to assume an interaction of BANF1 with both DNA transposons and retroviruses? In fact, BANF1 might be an ideal cellular factor for integrating elements in higher eukaryotes. Due to its non-specific DNA-binding activity to double-stranded DNA [58], a capacity to compact DNA and assemble higher-order nucleoprotein complexes, BANF1 could influence the fate of any extrachromosomal DNA molecule. As in retroviral integration [23], [69], BANF1 may compact the transposon genome to be a less accessible target for autointegration, and promote the integration step. Furthermore, similarly to retroviruses, BANF1 could be even actively recruited to preintegration complexes. The exact manner of recruitment might vary, providing specificity. BANF1 is recruited via physical interaction by the viral matrix protein *gag* to the retroviral preintegration complex of HIV-1 [62]. In *SB* transposition, BANF1 was enriched in a higher order complex containing the *SB* transposase and its interactor HMGXB4. Thus, the enrichment was mediated via protein-protein interaction. Since the experimental setup did not include the transposon DNA, we could not faithfully simulate preintegration complex formation. Nevertheless, HMGXB4 is a specific interaction partner of both the transposon and the transposase of *SB* [71]. Therefore, it might be reasonable to assume that BANF1 associates with the preintegration complex.

In sum, our strategy to model the process of establishing a host-transposon relationship in a naïve environment identified BANF1 as a host encoded factor influencing this process. Future work will have to clarify if a common role of BANF1 to protect integrating mobile elements in general exists.

### Excision, a step prior to integration, is already affected by the size of *SB* and *PB* transposons

Traditional models predict that efficient integration must follow the excision of DNA elements. Strikingly, autointegration was estimated to be over 90% in *mariner* transposition *in vitro*, suggesting that under the standard reaction conditions, the vast majority of the excised transposon inserts into itself, rather than into another DNA molecule [21]. This high frequency would establish autointegration as a major factor affecting productive integration. Furthermore, as longer transposons present more potential target sites, autointegration would be a reasonable explanation for size-dependence of transposition, observed for both *SB* [29], [30] and *PB* (this work) transposition.

Still, the role of autointegration in counteracting productive transposition might be overestimated. We found that transposon excision, a step prior to integration, is already affected by the size of the transposons (Figure 5A), indicating that a larger transposon might have difficulty to form a synaptic complex. Our data argue that competition between self-integration and productive transposition is unlikely to be the only factor responsible for sensitivity to size. If we assume that unproductive transposition equals suicidal autointegration, the gap between transposon excision and productive transposition could be a good estimate for the effect, and was reported to be around 25% in *SB* transposition *in vivo* [32].

### Excision, autointegration and transposition of *SB* and *PB* transposons are similarly affected by size

In contrast to an earlier report [41], we found that *SB* and *PB* transposons were affected similarly by the size of the transposon in three different assays (Figure 5). When the size of the transposon increased from 2683 to 7260 and 2795 to 7319 bps, the frequency of productive transposition dropped by 83% and 89.6% for *SB* and *PB*, respectively (Figure 4B). In addition, *SB* and *PB* behaved similarly in assays monitoring either excision or autointegration (Figure 5A). Therefore, our data argue against the general assumption that the *PB* transposon is not sensitive to size below 14 kb [41]. The different observation might be related to the fact that (i) the DNA fragment that Ding et al. used to increase the size of the transposon contained a higher density of TTAA target sites than the existing transposon. Actually, it is impossible to separate the true effects of length and numbers of target sites for a transposon that is highly specific in terms of integrating into a given sequence; (ii) Ding et al. estimated transposition frequencies in transgenic mouse experiments by counting transgenic embryos, regardless of the copy number of the integrated elements per embryo. Therefore, to compare productive transposition of *SB* and *PB* transposons, we have adjusted transgenic frequencies by the copy number of the integrated transgenes [72]. Importantly, small size does not seem to be an absolute requirement for mobilization in either case. Decreasing the distance outside the transposon ends of *SB* was reported to increase transpositional rates under experimental conditions [29]. Moreover, both *PB* and *SB100X* were reported to capable of mobilizing giant molecules of DNA, such as BACs (bacterial artificial chromosomes) [37], [73]. These reports indicate that in contrast to viruses, DNA transposons have no strict (if any) upper limit regarding their cargo capacity.

Autointegration of *SB*, likely due to physical constraints, avoided the IRs, suggesting that the captured events were rather intramolecular than intermolecular. Nevertheless, *SB* integration is not channelled to the terminal repeats of the transposon as it was observed for *Tn*10 [19]. Furthermore, the lack of linkage of autointegration sites to nearby regions at the donor DNA molecule would argue against an association between the ‘local hoping’ phenotype and autointegration.

### Aberrant transposition events may pose a threat to genome stability

Our experimental approach gave us the opportunity to have a closer insight into the mechanism of both *PB* and *SB* transpositions. We have captured autointegration products at comparable frequencies for both *SB* and *PB*. We assume that the excision and reintegration steps of autointegration and canonical transposition are mechanistically not significantly different [32], [41], [74] (Figures 1 and S1).

In addition to the autointegration products, our assays detected aberrant, pseudo-transposition events. In the ‘single-ended’ transposition products of *PB*, one IR of the transposon was clearly separated from the donor site, without obvious involvement of the other IR in the reaction (Figure 3D). The liberated end of *PB* targeted either the transposon or the backbone DNA (Figures 3C and 3E). *SB* did not display this feature in a similar assay system. By contrast, both transposons were capable of mobilizing substrates, lacking one of the IRs from separate molecules ([52] and this work). These bimolecular transposition events were eight-fold more frequently detected for *PB*.

How could aberrant transposition events be generated? In fact, ‘true single ended’ transposition, when a transposase interacts with a single transposon end, performs the cleavage and integration steps without the involvement of another end has not been undoubtedly reported from any system. In fact, alternative mechanisms can generate hard-to distinguish, similar products. For example, the canonical transposition reaction could fail at the final step, and only one end of the transposon is transferred (lariat model). In addition, our ‘solo’ experimental data support the ‘bimolecular model’, when the ends of the transposon derive from separate molecules [51]. In addition to single ended events, small deletions at the donor sites of *PB* transposition are assumed to be associated with imprecise transposon excision, and involve non-homologous end joining [40]. These structures were reported following *PB* excision in *Drosophila* (4.3%), mouse (5%) and in human cells [38], [40].

Aberrant pseudo-transposition can be considered as a fidelity problem of the transposition reaction, and has been observed with *P-element* in *Drosophila*, *Ds* element in *Arabidopsis*, Ac/*Ds* elements in maize [51], [75] or *Tam3* in *Antirrhinum majus* [76]–[80]. Small sequence variations generated by NHEJ at the excision sites are unlikely to cause genome rearrangements. By contrast, pseudo-transposition events can generate difficult-to-repair lesions and be genotoxic. Aberrant transposition events were reported to induce deletions, insertions, chromosome translocations and could initiate McClintock's chromosomal breakage-fusion-bridge cycles [51], [81]. Occasional mis-pairing between extrachromosomal molecules would not compromise the safety feature of a transposon-based transfer vector in a heterologous environment. However, fidelity problems could be problematic when the transposon is mobilized from the genome. Thus, cells subjected to *PB*-based genome manipulation techniques, e.g., transgene-free iPS cells generated by *PB* excision [82], should be carefully monitored for genome rearrangements.

### Wide host range vs fidelity: A price to pay?

There seems to be a basic difference in the ways transposons in pro- and eukaryotes control their activity to minimize the potential genotoxicity generated by improper synapsis of the transposon ends. For all classical bacterial transposons characterized to date, including *Tn*5 transposition, the catalytic steps of the reaction are tightly coupled to the synapsis of the transposon ends [83]. In addition, the coupling of transcription and translation in bacteria also increases the probability of a proper synapsis as the transposase binds tightly to the first IR before searching for nearby ends. In contrast, eukaryotic transposases must search at random for transposon ends when they enter the nucleus. Therefore, regulatory mechanisms promoting accurate double-ended reactions from the same transposon molecule are crucial.

*Tc1/mariner* transpositions, including SB, might have invented novel “built in regulatory checkpoints” to enforce synapsis prior catalysis [21]. A simple topological filter could also suppress promiscuous synapses of distant ends of the transposon [84]. Furthermore, certain transposition-like reactions, including V(D)J recombination, are also capable of filtering out unpaired reaction products. This regulatory mechanism, assisted by a cellular factor, HMGB1, regulates a highly controlled, ordered assembly process [85], [86]. Similarly to V(D)J recombination, HMGB1 was reported to assist paired end complex formation of *SB* [11]. In addition to HMGB1, *SB* transposition requires various vertebrate-specific host factors [10], [11], [13], [29] that render *SB* transposition restricted to vertebrates. In contrast, *PB* has an incredibly wide host range (from yeast to human) that could be associated with loose or no host factors requirement.

In comparison to *SB*, *PB* transposition results in more frequent, aberrant transposition products in a heterologous environment. Why is it so? If *PB* does not use host factors to enforce fidelity of the end pairing before excision, the reaction might be less precise by its nature. Alternatively, *PB* might utilize a host factor in its endogenous host (insect) that guarantees precise regulation. However, this factor is diverged or not available in mammalian cells. Finally, both *PB* and *SB* transposons have “built in regulatory checkpoints” that are most effectively filter out aberrant products under optimal conditions and in appropriate hosts. Notably, aberrant transposition events, including single-ended transposition of the *Mos1*, *mariner* element were observed under suboptimal conditions [22]. In sum, when a transposon is transferred too far from its original host, the conditions in a new environment could be suboptimal, and the fidelity of the reaction could be compromised. The wide host range of *PB* can be explained by relative independence from host-encoded factors, perhaps a price to be paid for fidelity.

## Materials and Methods

### Plasmid constructs

The IRs of the transposons were identical to the versions published earlier [49], [87] and were not modified for the assays. All the primers used for construct cloning were listed in Supporting Table S1. *SBrescue*: XmnI/BsaI fragment (Klenow-filled) containing ampicillin gene on pUC19 was replaced by PstI and SalI fragment containing zeocin gene from vector pZEO (isolate SV1, Invitrogen) resulting in *pUC19-zeo*. Klenow-filled SapI/SspI fragment containing zeocin gene and replication origin was inserted into EcoRI site of *PT2/HB* to get *PT2/SBzeo*. The transposon was PCR-amplified with primer AATASB-IR from *PT2/SBzeo* and ligated to rpsL gene fragment, which was PCR-amplified with primers rps1F/rpslR from nNG639 [43]. *SB2K*: BspHI/EcoRI fragment containing zeocin gene on *SBrescue* was replaced by BsaI/BglII fragment containing zeocin and promoter sequences from pFP-Zeo [88]. *SB3K, SB4K* and *SB7K*: DNA fragments were PCR-amplified from bacteriophage lamda DNA, using primers lam1kF/lam1kR, lam1kF/lam2kR and lam1kF/lam6kR, respectively, and were inserted into XbaI site (Klenow filled) of *SB2K*. *PB2K*: Klenow-filled NotI/HindIII fragment containing zeocin gene from *SBrescue* was inserted into SpeI site of pUC19PBneo [72] resulting in PUC19XLzeo. PvuII fragment containing *PB* transposon was ligated to rpsL gene PCR-amplified with primers rps1F/rpslR from nNG639. *PB3K, PB4K, PB7K*: The AatII/BglII fragments containing lamda DNA from *SB3K*, *SB4K* and *SB7K* were inserted into AatII/BglII sites of *PBPr* respectively. *pcDNA3.1BANF1* (BANF1 gene expressing vector): BANF1 coding sequence was PCR-amplified from pcDNA3.1/HiscBANF1 (a gift from Katherine Wilson, Johns Hopkins University) with primers BAFF/BAFR and cloned into EcoRV site of pcDNA3.1/Zeo (+) (Invitrogen). BAF-RNAi: Oligos of BAF96F/BAF96R were annealed together and cloned into BglII/HindIII site of pFP-Neo-H1 [88]. To generate ‘solo’ substrates *PB* pUC19XLneo [69] was digested with BamHI to delete the right IR (*PBΔright*) or with KpnI to remove the left IR (*PBΔleft*). For “solo” SB, *pTneo* was digested EcoRI to generate *SBΔleft*, while the digestion with BamHI yielded *SBΔright*.

### Cell culture maintenance and transfections

HeLa, AA8 and mouse MEF cells were cultured at 37°C with 5% CO_2_ in Dulbecco's modified Eagle's medium (DMEM, Gibco/Invitrogen) supplemented with 10% fetal calf serum (FCS, PAA). The zebrafish PAC2 cells were grown at room temperature and atmospheric CO_2_ concentrations in Leibovitz L15 medium (Gibco/Invitrogen) supplemented with 15% FCS. Cells were transfected at 50–80% confluence with QIAGEN-purified plasmid DNA using jetPEI (Polyplus transfection, for mammalian cells) or FuGene6 (Roche, for fish cells) according to instructions of manufacture. Transfection efficacy of a ∼3 kb and a ∼7 kb plasmid containing GFP cassette was monitored and compared by FACS analysis, but no significant difference was found (not shown).

### Autointegration assay

Cell culture and transfection was done as described [9]. Typically, 1.5×10^5^ cell were subjected to transfection with plasmids containing the transposon (500–1000 ng) and the transposase (60–100 ng). Two days post transfection plasmid DNA was recovered and transformed into bacteria (Invitrogen, ElectroMAX DH10B Cells, Cat. No. 18290-015, Genotype: F– *mcr*A Δ(*mrr*-*hsd*RMS-*mcr*BC) Φ80*lac*ZΔM15 Δ*lac*X74 *rec*A1 *end*A1 *ara*D139 Δ(*ara leu*) 7697 *gal*U *gal*K *rps*L *nup*G λ–). Bacteria were subjected to either zeocin (to determine total number of plasmids) or zeocin/streptomycin double selection (to determine autointegration events). The number of autointegration events was normalized by total number of plasmids. To confirm autointegration events, individual bacterial colonies were cultured and recovered plasmid DNA was subjected to DNA sequencing using primers of psbLacR3 and PB-F or PB-R for *SB-* and *PB* transposon, respectively. For BANF1 overexpression or knockdown experiments, 300 ng of pcDNA3.1BANF1 or BAF-RNAi plasmid was cotransfected with the transposon and helper constructs.

### Transposition assay

Cell culture and transfection was done as described [9]. Two days post transfection 10^5^ cells were plated on 10 cm dishes and exposed to antibiotic selection (100 ng/ml zeocin, for two weeks). Resistant colonies were visualized by methylene blue staining [9]. Transgene copy number was normalized by using qPCR specific to zeocin.

### Excision assay

The plasmid DNA was prepared as described in autointegration assay and dissolved in 50 µl water. Excision frequencies of eight transposon plasmid constructs of various sizes (four *SB* and four *PB*) were estimated by using a quantitative, real-time PCR (7700 sequence detection system from ABI, Applied Biosystems, Foster City, CA). To determine the total number of parental plasmid DNA molecules, a ‘parental’ titration curve was established. PCR primers of rpsL-F/rpsL-probe/rpsL-R were used to amplify the rpsL gene on the construct of *SBrescue*. For the curve, dilutions of 10^−2^, 10^−3^, 10^−4^, 10^−5^, 10^−6^ ng of *SBrescue* plasmid DNA were subjected to a PCR reaction to amplify the rpsL gene (rpsL-F/rpsL and probe/rpsL). To quantify the total number of parental plasmid molecules, total DNA extract was used (3 µl, diluted by 2000-fold, rpsL-F/rpsL-probe/rpsL-R). The excision products were PCR-amplified from the total extract DNA using nested PCR (1^st^ round, primers of rpslexciF1/rpslexciR1, 94°C for 30 s and 30 cycles of 94°C for 30 s, 58°C for 30 s, and 72°C for 30 s; 2^nd^ round, rpslexciF2/rpslexciR2, 1 µl, diluted by 100-fold, 94°C for 30 s and 35 cycles of 94°C for 30 s, 58°C for 30 s, and 72°C for 30 s). The amplified products (10^−2^, 10^−3^, 10^−4^, 10^−5^, 10^−6^ ng) were used to establish a second titration curve, specific for the excision products. To quantify excision products, primers of SB-F/SB-probe/SB-R and PB-F/PB-probe/PB-R were used on a total DNA extract (5 µl), for *SB* and for *PB*, respectively. The excision frequency was calculated as the ratio of excision products normalized by the total number of parental plasmid molecules. qPCR was performed for each experimental sample in triplicates. Ct values were determined following recommendations by the manufacturer.

### Colony PCR

Briefly, bacteria were picked by a pipette tip and directly subjected to a PCR assay using primers of PB-F and PB-R (5 pmol of each, Supporting Table S1) and *Taq* polymerase (Takara) in a total volume of 20 µl. PCR program: 94°C for 1 min; 30 cycles of 94°C for 30 s, 58°C for 30 s, and 2°C for 30 s; and 72°C for 2 min.

### Protein-protein interaction studies using the SILAC/pull-down assay

A triple SILAC pull-down experiment was performed using anti-HA resin. HEK293T cells were transiently transfected with HA-tagged wild type or mutant HMGXB4 (HMG2l1) [13] and SUMO1 in the presence/absence of *Sleeping Beauty*, SB10 [9] using Polyplus-transfection jetPEI transfection reagent with 3 µg of plasmids each. We compared proteins co-purifying with HA in cells expressing the empty vector (“light”), HA-tagged HMGXB4^−^ with mutated sumoylation site (“medium”) and HA-tagged wild-type HMGXB4 (“heavy”). The cells were plated on a 15-cm dish and harvested 48 h post-transfection. Two dishes were used for each condition. Detection of interaction partners is performed by mass spectrometry and the results obtained were analyzed by MaxQuant computational platform [89]. Results presented show protein abundance ratios between cells transfected with HMGXB4^−^ and the empty vector control.

### Co-immunoprecipitation, immunoblotting and antibodies

Whole-cell extracts were prepared using extraction buffer (Tris-HCl 50 mM at pH 8.0, NaCl 150 mM, 0.1% SDS (Na-dodecylsulphate) Triton X-100 1% and Na-deoxycholate 0.5%) supplemented with protease inhibitor cocktail (Roche, Mannheim, Germany). For immunoprecipitations, equal amounts of lysate (containing 5 mg of total cellular protein from HEK293 cells) were pre cleared with protein G-agarose beads (Sigma, St Louis, MO). Pre-cleared extracts were incubated with EZview Red Anti-HA Affinity Gel (Sigma-Aldrich, USA) for 1 h at 4°C. Precipitates were washed extensively in extraction buffer. Bound complexes were eluted with 2× SDS–PAGE sample buffer and resolved by 7.5–15% SDS–PAGE. Immunoblotting was performed according to standard procedures and proteins detected with the indicated antibodies. Antibodies were detected by chemiluminescence using ECL Advance Western Blotting Detection Kit (Amersham Bioscience).

## Supporting Information

Figure S1 Model of *PB* autointegration. The excision and reintegration steps of autointegration are similar to canonical transposition. For explanation see also Figure 3B. Similarly to *SB* autointegration products: (1) in the inversion products, the orientation of the IRs would be different from the donor substrate; (2) the inversion products would contain two ends of the transposon and target site duplications. Major differences to *SB*: (1) The target site is TTAA; (2) There is no footprint generated at the excision site, because the single stranded overhangs are simply ligated, precisely reforming the TTAA target site; (3) *PB* transposes via a hairpin intermediate, resolved by the transposase the excised transposon.
(TIF)

Figure S2 The ‘lariat’ model of single ended transposition. The canonical transposition reaction fails at the final step, and only one end of the transposon is transferred. The *PB* transposase-mediated events are targeted to TTAA, and can be clearly distinguished from non-specific recombination events. The liberated single IR attacks the target site, TTAA either on the backbone or on the transposon DNA. The polarity of the reaction is reflected by the position of the targeted TTAA. Products of I, III or II, IV would be detectable by using constructs *PB2K* and *PBsingle*, respectively. *PB* transposon (gray), donor DNA (black). Note: In addition to the “lariat” model similar products could be generated by alternative mechanisms (see text).
(TIF)

Figure S3 Knockdown of BAF1 by RNA interference. The knock-down effect of the RNAi approach [88] used against BANF1 monitored by Western blotting (25 µg of total cell lysates). Actin was to monitor for equal loading.
(TIF)

Figure S4 The cellular factor, HMGB1 does not affect *SB* autointegration. Relative autointegration frequencies of *SB* (*SB7K*) in HeLa cells, where HMGB1 was either knocked-out [60] (HMG-) or overexpressed (cHMG). No significant effect was detected in either case when compared to the wild type (100%).
(TIF)

Table S1 Primers sequences.
(DOCX)

Text S1 Transposition of ‘solo’ substrates, lacking one of the IR of the transposon.
(DOCX)
