# Correction: Suicidal Autointegration of *Sleeping Beauty* and *piggyBac* Transposons in Eukaryotic Cells

**DOI:** 10.1371/journal.pgen.1008406

**Published:** 2019-09-23

**Authors:** Yongming Wang, Jichang Wang, Anatharam Devaraj, Manvendra Singh, Ana Jimenez Orgaz, Jia-Xuan Chen, Matthias Selbach, Zoltán Ivics, Zsuzsanna Izsvák

The following information is missing from the Funding statement: ZI is funded by European Research Council-2011-ADG-TRANSPOSOstress—294742.
