# Supplementary figures and images for: Suicidal Autointegration of Sleeping Beauty and piggyBac Transposons in Eukaryotic Cells
Source: PLoS Genet. 2014 Mar 13;10(3):e1004103. doi: 10.1371/journal.pgen.1004103 (PMC3952818; doi:10.1371/journal.pgen.1004103)

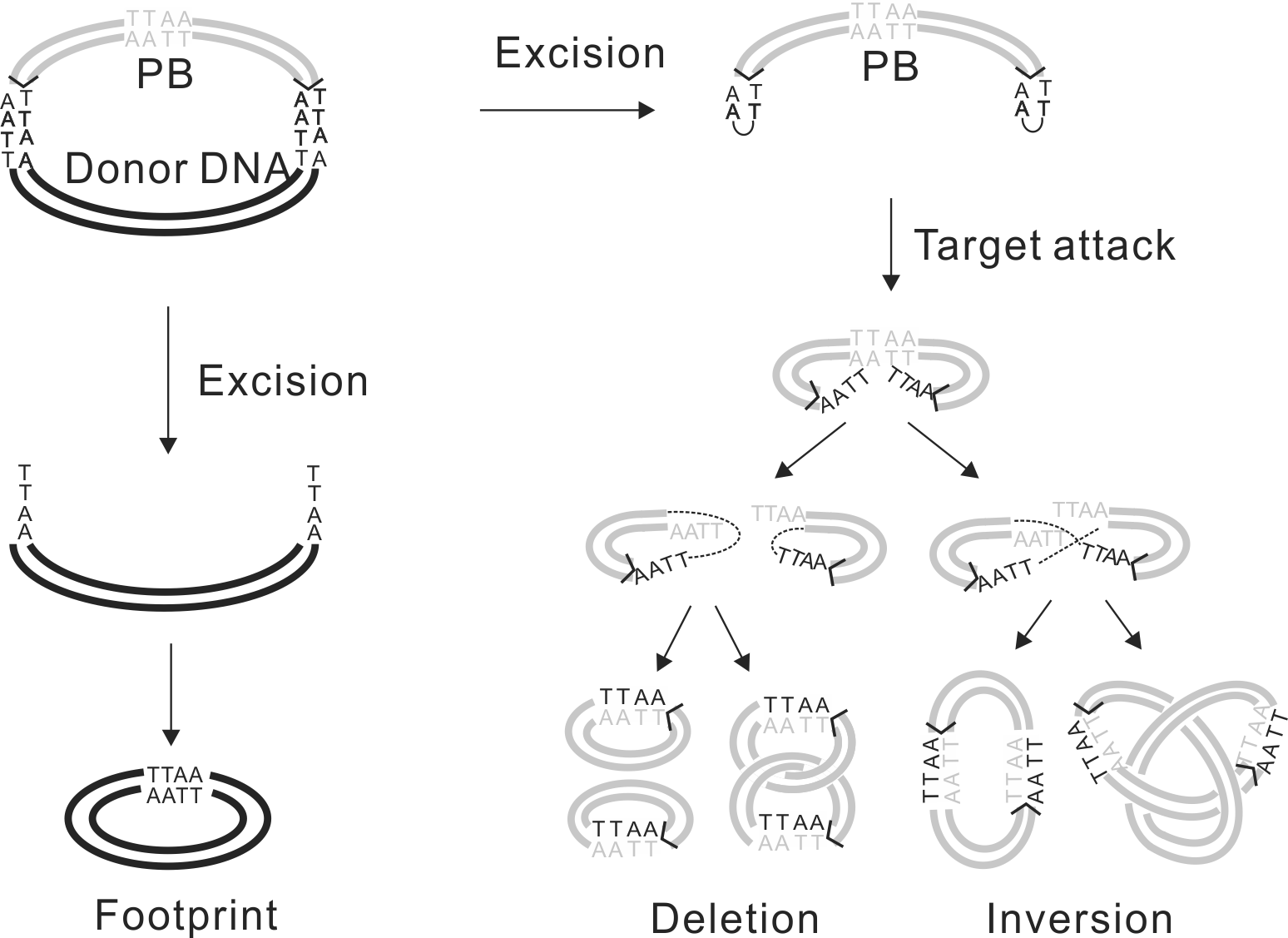

Supplement: Figure S1 — Model of PB autointegration. The excision and reintegration steps of autointegration are similar to canonical transposition. For explanation see also Figure 3B. Similarly to SB autointegration products: (1) in the inversion products, the orientation of the IRs would be different from the donor substrate; (2) the inversion products would contain two ends of the transposon and target site duplications. Major differences to SB: (1) The target site is TTAA; (2) There is no footprint generated at the excision site, because the single stranded overhangs are simply ligated, precisely reforming the TTAA target site; (3) PB transposes via a hairpin intermediate, resolved by the transposase the excised transposon. (TIF) [file pgen.1004103.s001.tif]

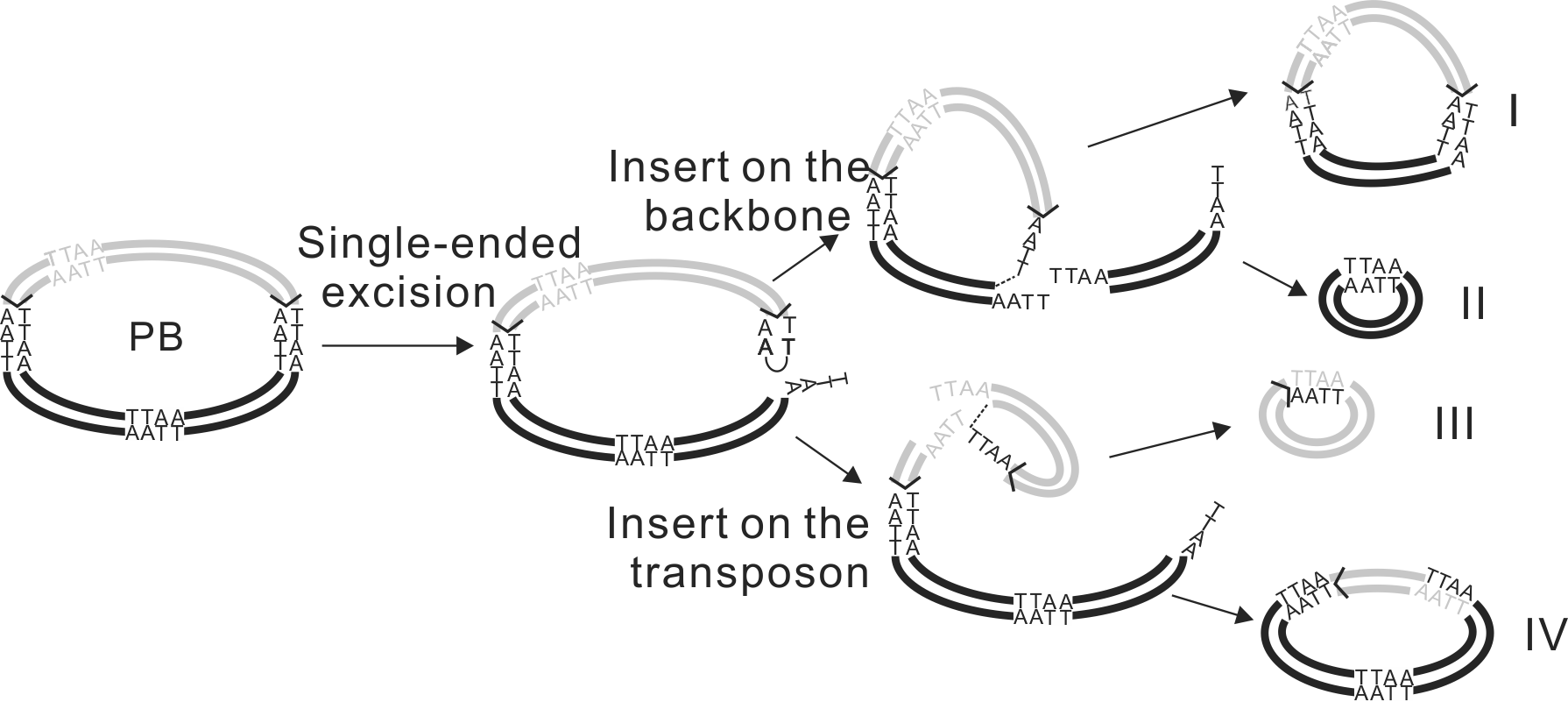

Supplement: Figure S2 — The ‘lariat’ model of single ended transposition. The canonical transposition reaction fails at the final step, and only one end of the transposon is transferred. The PB transposase-mediated events are targeted to TTAA, and can be clearly distinguished from non-specific recombination events. The liberated single IR attacks the target site, TTAA either on the backbone or on the transposon DNA. The polarity of the reaction is reflected by the position of the targeted TTAA. Products of I, III or II, IV would be detectable by using constructs PB2K and PBsingle, respectively. PB transposon (gray), donor DNA (black). Note: In addition to the “lariat” model similar products could be generated by alternative mechanisms (see text). (TIF) [file pgen.1004103.s002.tif]

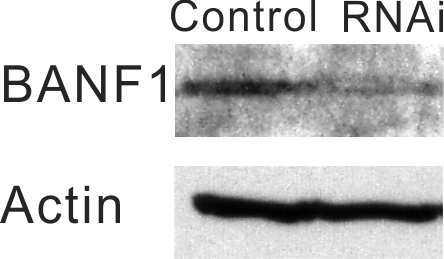

Supplement: Figure S3 — Knockdown of BAF1 by RNA interference. The knock-down effect of the RNAi approach [88] used against BANF1 monitored by Western blotting (25 µg of total cell lysates). Actin was to monitor for equal loading. (TIF) [file pgen.1004103.s003.tif]

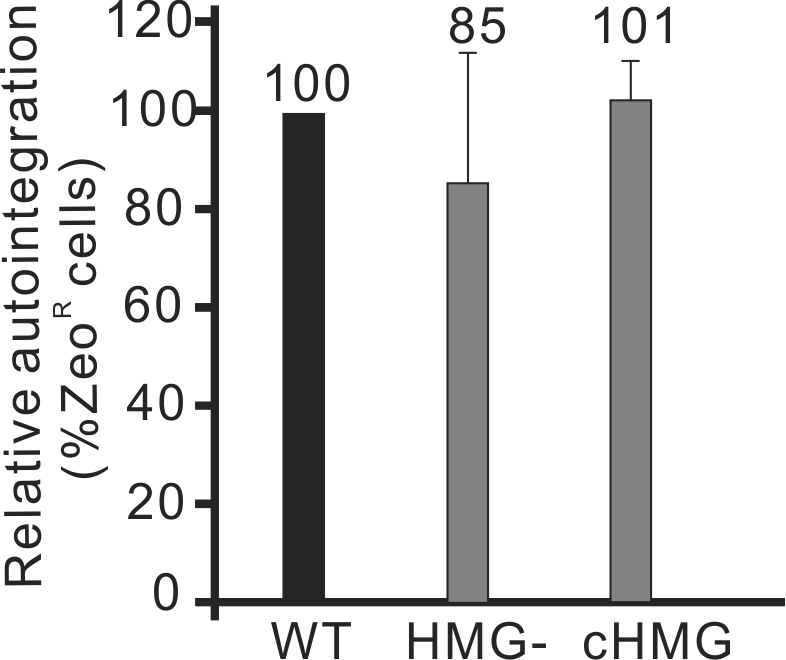

Supplement: Figure S4 — The cellular factor, HMGB1 does not affect SB autointegration. Relative autointegration frequencies of SB (SB7K) in HeLa cells, where HMGB1 was either knocked-out [60] (HMG-) or overexpressed (cHMG). No significant effect was detected in either case when compared to the wild type (100%). (TIF) [file pgen.1004103.s004.tif]
