# Supplementary material for: Suicidal Autointegration of Sleeping Beauty and piggyBac Transposons in Eukaryotic Cells
Source: PLoS Genet. 2014 Mar 13;10(3):e1004103. doi: 10.1371/journal.pgen.1004103 (PMC3952818; doi:10.1371/journal.pgen.1004103)
Supplement: Table S1 — Primers sequences. (DOCX) [file pgen.1004103.s005.docx]

Table S1.1 **Primers for construct cloning and colony PCR**

| AATASB-IR | AATACAGTTGAAGTCGGAAGTTTACA |
| --- | --- |
| rps1F | CCCAAGCTTGATCTTACGGTTAAGCACCC |
| rpslR | ACGGCAGGTATATGTGATGGGT |
| psbLacR3 | TACCAAATACTAATTGAGTG |
| lam1kF | GCAAATGTCATCGACGTTTTTA |
| lam1kR | CGGAAGTTAACGCTAAAGCACT |
| lam2kR | GAATGAGTACTGCACTCGCAAC |
| lam6kR | ATTGGTTTTGCCAGAACTGTTT |
| BAF-96F | GATCCCCGAAGCTGGAGGAAAGGGGTTTCAAGAGAACCCCTTTCCTCCAGCTTCTTTTTGGAAA |
| BAF-96R | AGCTTTTCCAAAAAGAAGCTGGAGGAAAGGGGTTCTCTTGAAACCCCTTTCCTCCAGCTTCGGG |
| PB-F | CTTTCCTACTTGGCAGTACATCTAC |
| PB-R | TTCAGGGTCAGCTTGCCGTAGGTGG |

Table S1.2 **Primers for Taqman PCR in excision assay**

| rpsL-F | AAGCACCCCAGCCAGATG |
| --- | --- |
| rpsL-probe | CCTGGTGATGGCGGGATCGTTGTA |
| rpsL-R | TGCCGAAAAGGTGTCAAGAAA |
| SBPr-F | CCCATCACATATACCTGCCGTAA |
| SBPr-probe | TACGGTTAAGCACCCCAGCCAGATGG |
| SBPr-R | CAACGATCCCGCCATCAC |
| PB-F | TGACCATGATTACGCCAAGCT |
| PB-probe | CGAGCTCGAATTCACTGGCCG |
| PB-R | CCCAGTCACGACGTTGTAAAAC |
| rpslexciF1 | TACATCGGTGGTGAAGGTCA |
| rpslexciF2 | AAGCAGGCTCGTTCCAAGTA |
| rpslexciR1 | ACGTTGCTTTTCGCAACTTT |
| rpslexciR2 | GCCGAAAAGGTGTCAAGAAA |

Table S1.3 **Primers determining the other, IR deleted end of the *PB* transposon (Clone #8)**

| Neo3’#1 | CGTTGGCTACCCGTGATATT |
| --- | --- |
| Neo3’#2 | TCGCCTTCTTGACGAGTTCT |
| 2_gDNA#1 | CCGCAGAAAAGTCAACTTCC, Chr3 149971374, strand + |
| 2_gDNA#2 | ACGTGTGAACACAGCCCTCT 3' Chr3: 149971398, strand + |
