# Supplementary material for: Suicidal Autointegration of Sleeping Beauty and piggyBac Transposons in Eukaryotic Cells
Source: PLoS Genet. 2014 Mar 13;10(3):e1004103. doi: 10.1371/journal.pgen.1004103 (PMC3952818; doi:10.1371/journal.pgen.1004103)
Supplement: Text S1 — Transposition of ‘solo’ substrates, lacking one of the IR of the transposon. (DOCX) [file pgen.1004103.s006.docx]

Integration sites were analysed by linker-mediated PCR {Ivics, 2011 #479}{Grabundzija, 2010 #480}.

Examples of transposase-mediated integration of *PB*Δ*left (*hg19 assembly) positions define the first T of the target site TTAA.

(Blue letters indicate the right ITR sequence, the black letters indicate gDNA):

1. GACCGATAAAACACATGCGTCAATTTTACGCATGATTATCTTTAACGTACGTCACAATATGATTATCTTTCTAGGGTTAACACTGCTTTAGGGTGTCCCAGAGATTCTGGAATGTTGTCTCTTTGTTCTCATTGGTATCAAAGAACTTACTGATTTCTGCCTTAATTTCCTAATTTACACAGGAGTCATTCAGGAGCAGGTTGTTCAATGTCCATGTAATTGATTTGTTTTGAGTGAGTTTCTTAATCCTGAGTTCTAATTTTATTGCACTGTGGTCTAAGAGACTGTTTGTTACAGTTTCCATTATTTTGCATTTGCTGAGGAGTGTTTTACTTCCAATTACATGGTCAATTTTAGAATAAGTGCCATGTGGCACTAAGAATATCCTTGTTAATTTTCTGTCTAATATTGACAGTGGGGTGTTAAAATCTCCCATTATTTTTGTGTGGGAGTCTAAGTCTCTTCGTAGGTCTCTAAGAACTTGTTTTATGAATCTGGGTGCTCTTGTATTGGGTGCATATATATTTAGAATAATT Chr5:144,751,665144751665 strand +
2. GACCGATAAAACACATGCGTCAATTTTACGCATGATTATCTTTAACGTACGTCACAATATGATTATCTTTCTAGGGTTAAGTATAGTTTGGTGGGCAGGGGACTAGGAAATGGGGAATGTTGAAGAATAAATCACAGGGAGTTGA

Chr11:72,281,299 strand -

1. CTGGCTATTTTGTGTACGCAGTTAAAATGCGTACTAATAGAAATTGCATGCAGTGTTATACTAATAGAAAGATCCCAATTTTTGATGTATTAGAATAAATTACCTGCAATAAAGAAAAGAGGTCAAACAGACCTGGAAT

Chr6:64227195 strand +

1. CTGGCTATTTTGTGTACGCAGTTAAAATGCGTACTAATAGAAATTGCATGCAGTGTTATACTAATAGAAAGATCCCAATTTTGGCATCAAGGACAAACCCACCTTCATCAAAGGGGTGAGCAGTTCCCCATGCCAGGTCGTGCTAAAGAAGTGTTTGTTGGCTGGGTGCAGTGGTTCGCACCTGTAATCCCAGCACTTTGGGAGGCCAAGGTGGGTGGATCACCTGAGGTATGGAGTTTGAGACCAGCCTGGCCAACATGGTGAAACCCCGTCTCTACTAAAAATACAAAAATT

Chr11:47,238,562 strand +

1. GACCGATAAAACACATGCGTCAATTTTACGCATGATTATCTTTAACGTACGTCACAATATGATTATCTTTCTAGGGTTAAAGTATATGGACATTGTGGAAAGATGTTTTTCCCCCATATGGCTTCTCCTCCCTTTTGCCCTGGGCCCTGAAAGCCTGAAGTAGACTCCCACACTCTTTCTCTCCCTCTCTGTTCTCAGAAATCTCCAGACATATATTTGAAGATAAACCAACAAACACAGACATTTCATTTGTTTAGCAAACCGAAAGGTTACGTTTGGGGGGCTGTTCAAGTCATACTCTACCCACTTAACTTTCTGCAGTCTCCAAAATAGAGTCAATCTGTAATATCTTACAAAGTCAGAGTGACTT

chr10:74,004,149 hg19 assembly, strand +

1. GACCGATAAAACACATGCGTCAATTTTACGCATGATTATCTTTAACGTACGTCACAATATGATTATCTTTCTAGGGTTAATGGATTTGGCACTGGAAAAAAACTCTTATGTTGCATTCTTATCTGTTATCAGTGGTTATTTCATAGGCCAAGTGTTCCTGTGGTTGGGCAGGGTGTAACACAACATA

Chr18: 12373292, strand -

1. GACCGATAAAACACATGCGTCAATTTTACGCATGATTATCTTTAACGTACGTCACAATATGATTATCTTTCTAGGGTTAAAGAACACCATCCACCCAGAGGGCA

Chr7:155,308,901, strand +

1. GACCGATAAAACACATGCGTCAATTTTACGCATGATTATCTTTAACGTACGTCACAATATGATTATCTTTCTAGGG*TTAA*TATAAATATTACCAACTCCATATCAGTCTTAATTTTAATAATAACGCCAAACAATGGATTCTTTTCTGTCACGTTTTGCAATATAATCATGTCAGTTTTCAAAGGCTGGTACACCCTGGAATTACAAACACAGTCTGTGGCAAGAGATATTGGC

Chr3: 149971417, strand +


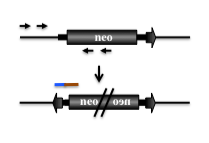


**Figure. Analysis of clone #8 (*PB*Δ*left).*** Clone #8 (*PB*Δ*left)* was further analysed to determine the structure of the other, IR deleted end of the transposon. Using primers designed to genomic sequence and to the *neo* gene the sequence of second right IR was identified by PCR in an inversed orientation (for primers see Supplementary Table S1). The double lines indicate recombination. The colored line corresponds to the sequence information shown below:

GTGACGNACGTTNANATAATCATGTGTAAAATTGACGCATGTGTTTTATCGGTCTGTATATCGAGGTTTATTTATTAATTTGAATAGATATTAAGTTTTATTATATTTACACTTACATACTAATAATAAATTCAACAAACAATTTATTTATGTTTATTTATTTATTAAAAAAAACAAAAACTCAAAATTTCTTCTATAAAGTAACAAAACTTTTATCGAATTCCTGCAGCCCGGGGGATCCACTAGTAGTCAATTCTGTGGAATGTGTGTCAGTTAGGGTGTGGAAAGTCCCCAGGCTCCCCAGCAGGCAGAAGTATGCAAAGCATGCATCTCAATTAGTCAGCAACCAGGTGTGGAAAGTCCCCAGGCTCCCCAGCAGGTAGAAGTATGCAAAGCATGCATCTCAATTAGTCANCAACCATA (matches neo)

Examples of transposase-mediated integration of *SBΔright*

1. CCAGGTAGGAGTTGGCAACTATTGTTTGTTACAAGAAATTTGTGGAGTAGTTGAAAACGAGTTTTAATGCCAGATATAAAAGGGGTAAGTGTATGTAAACTTCCGACTTCAACTGTAGAATCTTTTTAACTTCAAAATTCATATTCGGGCTCTTTACGTCCTTGAGTCTTTTGGCCCATATCACCTTCCACCTCATTCATTGTGAACAAAGCTCAATCAGAAATCTTTGTGCTCTTTTCTGGAAGTGTGGAAGGCAATTTTCAGCAAAATCATTAACTAGAAGGGGGAAATGGTTTTTTTGAATGTGTATAATAGACCTCTACAAACAACACGAAGCAGAATATATTTGAAAAACAGTT

Chr2: 180406656, strand -

1. AAAACGAGTTTTAATGACTCCAACTTAAGTGTATGTAAACTTCCGACTTCAACTGTATTTAAGAGTTTGAATGTGCTGAGTGGATGCTTTGGAAACCTAACCTCAGGCAAAAGGTCCTTGGTTCAGGCTTGGTGACTGATGTTGCATTGTCTGCTGTTAATAGCCTAGAGATTGAAGACCCAAGTAGGCCATATTTTTGCCTGTCAACAGCGAATCTCCAATCTTTAACTTCTTTAGTTTCAATATTTGATTTTGCTTGGGTTCACCTCTACAGTTGCTTTGCCTTGTCCCTGGTCACCTTACTATATTTGACTGAATTAAGCTTTCTGTTTCAATGTAAGTACTTAGTATTTTGCATTTAAAAGGCTGTTCTTTAATAACTTTGGAGGAAGTTGACTGATTTTAATGAAATTCTGTCCATTATAAAGGCATTCACATACACCTCAGGTTTAATTTAAATGCAGTCTGTCCCTTGAAATGAAATGGCAATGGGCAAAAGTCTT

Chr10: 77238001 strand +

1. AATGAAATTCAAACATTTCGTTATACAGAAATTTGTGGAGAGTTGAAAACGAGTTTTATGACCTCCAACATTAAGGTGTTTGTAACTTCCGACTTCAACTGTAGGGGACCCCTCTT

Unidentified genomic locus

1. TTAACATTGCCAAACTATTGTTTGTTACAGAAATTTGTGGAGTAGTTGAAAAACGAGTTTTAATGCCTCCAACTTAAGTGTATGTAAACTTCCGACTTCAACTGTACATGAATTGAGAAAAAAATATCAATATTCTTGATGATAACCATCGCCAAACAAGTTTAAAGATACAGGACTTTAAAGTGAACCTACAAGAATAAATACCAAGTAACTAACAACGGAGTCAGAAAAACGTGCCAGGCACAGGGGGGTGTGCCTGTGGTCCACAGCTACTCGGGAGGCTGAGGCAG

Chr3:189595933 strand –
